# Unravelling genetic variation underlying *de novo*-synthesis of bovine milk fatty acids

**DOI:** 10.1038/s41598-018-20476-0

**Published:** 2018-02-01

**Authors:** Tim Martin Knutsen, Hanne Gro Olsen, Valeria Tafintseva, Morten Svendsen, Achim Kohler, Matthew Peter Kent, Sigbjørn Lien

**Affiliations:** 1 Centre for Integrative Genetics (CIGENE), Department of Animal and Aquacultural Sciences (IHA), Faculty of Life Sciences (BIOVIT), Norwegian University of Life Sciences (NMBU), PO Box 5003 Ås, Norway; 2 Faculty of Science and Technology (RealTek), Norwegian University of Life Sciences (NMBU), PO Box 5003 Ås, Norway; 3 Geno Breeding and AI Association, N-1432 Ås, Norway

## Abstract

The relative abundance of specific fatty acids in milk can be important for consumer health and manufacturing properties of dairy products. Understanding of genes controlling milk fat synthesis may contribute to the development of dairy products with high quality and nutritional value. This study aims to identify key genes and genetic variants affecting *de novo* synthesis of the short- and medium-chained fatty acids C4:0 to C14:0. A genome-wide association study using 609,361 SNP markers and 1,811 animals was performed to detect genomic regions affecting fatty acid levels. These regions were further refined using sequencing data to impute millions of additional genetic variants. Results suggest associations of *PAEP* with the content of C4:0, *AACS* with the content of fatty acids C4:0-C6:0, *NCOA6* or *ACSS2* with the longer chain fatty acids C6:0-C14:0, and *FASN* mainly associated with content of C14:0. None of the top-ranking markers caused amino acid shifts but were mostly situated in putatively regulating regions and suggested a regulatory role of the QTLs. Sequencing mRNA from bovine milk confirmed the expression of all candidate genes which, combined with knowledge of their roles in fat biosynthesis, supports their potential role in *de novo* synthesis of bovine milk fatty acids.

## Introduction

Bovine milk is an important source of many nutrients including proteins, fat, minerals, vitamins and bioactive lipid components. The relative abundance and concentration of individual fatty acids (FAs) in milk affect both human health and the manufacturing properties of dairy products. Myristic (C14:0) and palmitic acid (C16:0) are associated with cardiovascular disease through increased level of blood cholesterol^1^, while shorter chain saturated FAs (C4:0 to C12:0) have been associated with positive health effects such as antiviral, antibacterial and anticancer activities^2–4^. The difference in melting point between saturated and unsaturated acids also affects the softness, flavour and colour of dairy products such as butter and cheese^5,6^.

By improving our understanding of the pathways in bovine milk FA synthesis and identifying the genes and genetic polymorphisms associated with variation in milk FA content, it may be achievable through genome-based selection methods^7^ to optimally balance individual FAs allowing industry to satisfy consumer demands for healthy food of high quality. The short- and medium-chain length acids C4:0 to C14:0 are potential targets for this purpose. In contrast to the bulk of long-chained milk FAs and around half of C16:0 which are largely derived from the cow’s diet, C6:0 to C14:0 and a fraction of C4:0 are synthesized *de novo* in the bovine mammary gland^8^. These acids occur in milk in relatively high concentrations and show moderately high heritabilities (usually in the range of 0.10 to 0.50)^9–12^ and are therefore well suited for genetic analyses such as a genome-wide association study (GWAS).

The synthesis of short- and medium-chained FAs is founded upon C2 and C4 precursors absorbed from the diet. After being transported to the mammary gland, acetate and acetoacetate are converted to acetyl-CoA and then to malonyl-CoA which, along with butyryl-CoA (from plasma β-hydroxybutyrate and C2), are used as precursors for cytosolic *de novo*-synthesis. The process of carbon chain elongation from C2:0 or C4:0 to C16:0 involves a cyclic reaction^13^ which also generates intermediate products, C4:0 to C14:0, via a chain termination mechanism^14^. Newly synthesised FAs are transported from the cytosol to the endoplasmic reticulum where they are linked to a glycerol 3-phosphate backbone to form triacylglycerols, a final series of steps sees them secreted into the milk in the form of milk fat globules.

The current study explores genetic variation associated with the *de novo*-synthesis of short- and medium-chained FAs (C4:0 to C14:0). Milk fatty acid composition was predicted from Fourier transform infrared spectroscopy (FTIR) using prediction equations derived from GC/FTIR calibration sets. This method has been shown to provide fast and cheap large-scale phenotyping of the breeding population, especially for acids with relatively high concentration and heritability such as the *de novo*-synthesized FAs^12,15–17^. These phenotypes were combined with array-based single nucleotide polymorphism (SNP) genotypes in a genome-wide association study to identify chromosomal regions (quantitative trait loci - QTLs) with substantial effects on the traits under investigation. QTL regions identified on bovine chromosomes (BTA) 11, 13, 17 and 19 were re-analysed using a higher density of sequence variants (SNPs and indels) imputed from re-sequencing data in an attempt to identify putative functional polymorphisms. Moreover, mRNA sequence analysis of mammary epithelial cells from 36 milk samples was used to verify that the candidate genes indicated by GWAS were expressed in the mammary gland during milk production.

## Results

### Genome-wide association analyses for FA concentration

Our analysis began with combining daughter yield deviations (DYDs) for C4:0 to C14:0 from 1,811 bulls with genotypes from 609,361 autosomal SNPs to perform a GWAS and identify chromosomal regions with a major impact on *de novo* synthesis of these acids.

**Figure 1:**
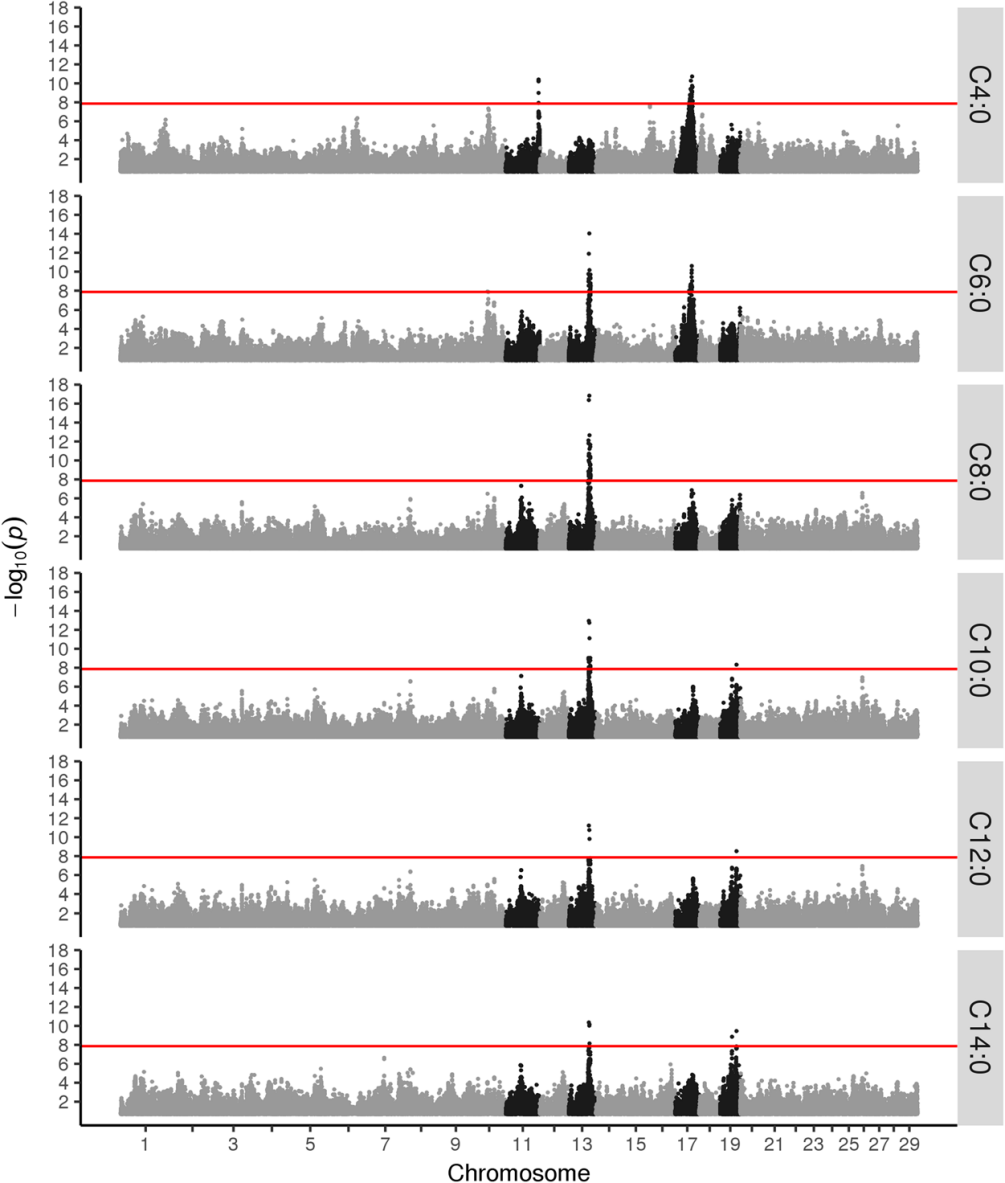
Manhattan plots showing results from genome-wide association analyses of C4:0 to C14:0 on high-density marker data. Chromosomes are shown along the abscissa while the ordinate denotes the −log_10_(p-value) for each marker – trait association. Chromosomes showing genome-wise significant associations for one or more of the tested acids are highlighted with black points. The red line denotes the genome-wide significance level.

As shown in Fig. 1, we found the most significant associations on BTA11, BTA13, BTA17 and BTA19. Results for all significant marker and trait combinations are provided in Supplementary Table S1. The QTL on BTA11 was most significant for the shortest of the tested acids; C4:0, while the one on BTA17 was significant for both C4:0 and C6:0. As FA chain length increases, these regions become less important, while the significance of the QTL on BTA13 increases. This QTL was most significant for acids with intermediate chain lengths (especially C8:0) with decreasing significance for shorter (C6:0) and longer acids (C10:0–C14:0). Finally, the QTL on BTA19 becomes the most significant for the synthesis of the longest of the analysed acids; C14:0.

All major QTL regions spanned genes with an established function in milk fat biosynthesis. The QTL on BTA11 was detected close to the *associated endometrial protein* (*PAEP*) gene at 103.3 Mb. The QTL region on BTA13 was rather broad and covered at least two potential candidate genes; *nuclear receptor coactivator 6* (*NCOA6*) at 64.6 Mb and *acyl-CoA synthetase short-chain family member 2* (*ACSS2*) gene at 64.8 Mb. BTA17 displayed a QTL close to the *acetoacetyl-CoA synthetase* (*AACS*) gene at 53 Mb. Closer examinations of BTA19 revealed that the associations were located in two distinct regions; one at 37.4 Mb which is around 500 kb from *acyl-CoA synthetase family member 2, mitochondrial precursor* (*ACSF2*) at 36.9 Mb. The second QTL region was close to *fatty acid synthase* (*FASN*) at 51.4 Mb. However, analysis of sequence variants revealed that the significant markers detected around 36.9 Mb were not situated within or very close to *ACSF2*. Since no other convincing candidate gene was detected in this region, we chose not to follow up this QTL in further analyses.

### Fine-mapping of imputed sequence variants on selected chromosomes

To characterize as much genetic variation as possible in and around the candidate genes we imputed SNPs and indels identified from whole genome sequence data resulting in a more than 20-fold increase in marker density after quality filtering in the regions 90–110 Mb on BTA11, 60–70 Mb on BTA13, 20–60 Mb on BTA17, and 45–55 Mb on BTA19. The quality of imputation relates most to marker allele frequencies. The Beagle software^18^ reports an internally calculated parameter, allelic r-squared (AR^2^), that is the estimated squared correlation between the most likely allele and the true allele for each marker^19^. The mean value of this parameter ranged from 0.84 for variants with minor allele frequency (MAF) below 0.05 to 0.94 for variants with MAF above 0.05. Imputed SNPs and indels, with AR^2^ above 0.7, were included in a reanalysis of the QTLs for significant phenotype associations. Detailed information of the top significant variants on each chromosome for each FA tested, is shown in Table 1.

**Table 1:** Summary of the top variants on chromosomes 11, 13, 17 and 19 determined by single-marker association analyses of sequence variants for fatty acids C4:0 to C14:0.

FA	BTA	rs number	Top variant (bp)	Ref allele	Alt allele	MAF	p-value
C4:0	11	rs109837926	103,300,697	C	A	0.34	3.47e-9
C4:0	13	—	62,280,697	A	G	0.13	7.37e-7
C4:0	17	rs477658921	53,078,216	GAAAGTGA	G	0.08	8.09e-11
C4:0	19	rs797503644	52,884,766	G	A	0.28	2.17e-8
C6:0	13	rs41700742	64,648,620	A	G	0.45	6.82e-16
C6:0	17	rs379029510	51,669,903	T	C	0.46	2.05e-10
C6:0	19	rs476079746	37,421,626	G	GAAAAAA	0.40	5.86e-9
C8:0	13	rs381037433	64,523,817	G	GA	0.21	1.08e-18
C8:0	17	rs456738710	51,161,184	A	T	0.22	1.31e-8
C8:0	19	rs457952543	51,334,328	C	CT	0.03	1.26e-6
C10:0	13	rs381037433	64,523,817	G	GA	0.21	7.6e-16
C10:0	17	rs456738710	51,161,184	A	T	0.22	2.47e-8
C10:0	19	rs109016955	51,381,233	G	C	0.04	5.21e-9
C12:0	13	rs381037433	64,523,817	G	GA	0.21	4.79e-14
C12:0	17	rs456738710	51,161,184	A	T	0.22	2.17e-7
C12:0	19	rs109016955	51,381,233	G	C	0.04	7.5e-9
C14:0	13	rs381037433	64,523,817	G	GA	0.21	5.2e-14
C14:0	17	rs384370770	51,231,279	T	C	0.09	3.67e-8
C14:0	19	rs109016955	51,381,233	G	C	0.04	4.07e-11

FA, fatty acid; BTA, *bos taurus* chromosome; rs, rs number; Top variant, position of the most significant markers in base pairs; Ref allele, reference allele; Alt allele, alternative allele; MAF, minor allele frequency.

### Chromosome 11

**Figure 2:**
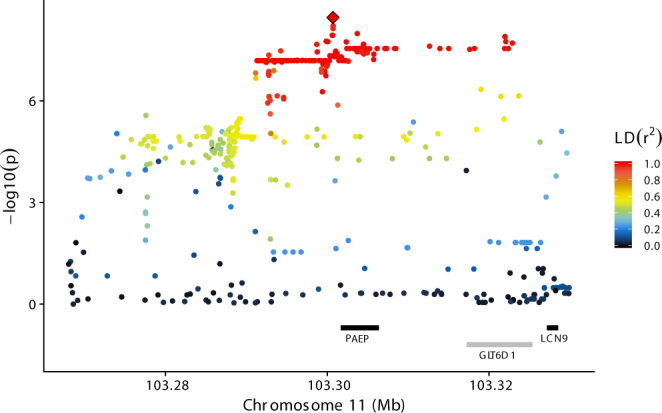
Results for BTA11 - C4:0 association analysis using imputed sequence variant data on BTA11, zoomed in on the region between 103.27 and 103.33 Mb. The ordinate provides −log_10_(p-value) for each marker – trait association, while the abscissa denotes marker position. The red diamond indicates the most significant marker for C4:0; rs109837926 at position 103,300,697 bp. Colouring indicates the level of LD (r^2^) between each marker and rs109837926. Gene annotation information (Ensembl Bos taurus annotation release 86) is shown with grey and black bars reflecting positive and negative strand orientations respectively.

Results for BTA11 showed the strongest associations between C4:0 and a group of markers that were situated within and immediately outside of *PAEP* (Fig. 2). The most significant marker (rs109837926; p-value = 3.5e-9) was found at position 103,300,697 bp which is ≈800 bp upstream from *PAEP*’s transcription start site. The minor A allele (MAF = 0.34) was associated with a slight, but noteworthy increase in C4:0 levels (0.02 g/100 g milk fat). Closer examination of the haplotype containing the top ranked markers (all of which had p-values, effects and frequencies similar to rs109837926) revealed that the minor alleles for all markers were included in a single haplotype (frequency ≈ 0.3) that covered a region beginning 11 kb upstream from *PAEP* and extending into the neighbouring gene *glycosyltransferase 6 domain containing 1* (*GLT6D1*). The high level of linkage disequilibrium (LD) among these markers (Fig. 2) restricted our ability to pinpoint any one of them as causal. Among other top-ranking markers were two missense variants in *PAEP*, known to produce the A and B protein variants of beta-lactoglobulin (at 103,303,475 bp in exon 3 and 103,304,757 bp in exon 4), one splice region variant (at 103,304,656 bp in *PAEP* exon 3), and three SNPs in the 5′UTR of *PAEP* (103,301,561 bp, 103,301,690 bp, and 103,301,694 bp). The four top-ranked markers were grouped in a region ≈1,000 bp upstream of *PAEP* which can suggest a regulatory role of the QTL. Results for all tested markers and trait combinations are shown in Supplementary Fig. S6 and Supplementary Table S2.

### Chromosome 13

**Figure 3:**
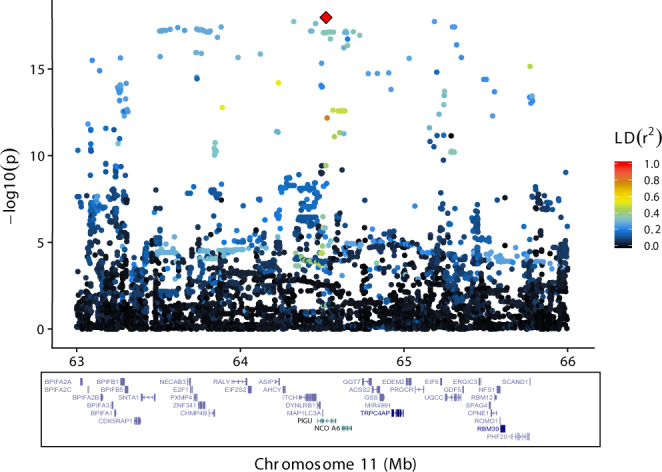
Results for BTA13 - C8:0 association analysis using imputed sequence variant data on BTA13, zoomed in on the region between 63 and 66 Mb. The ordinate provides −log_10_(p-value) for each marker – trait association, while the abscissa denotes marker position. The red diamond indicates the most significant marker for C8:0; rs381037433 at position 64,523,817 bp. Colouring indicates the level of LD (r^2^) between each marker and rs381037433. Gene annotation information (Ensembl Bos taurus annotation release 86) is shown with blue bars reflecting position and exon structure.

On BTA13, the most significant results were found for C8:0, with decreasing significance levels for acids with shorter and longer chain lengths. For all traits, we detected similar p-values for a large number of markers in a region spanning from approximately 63.5 to 65.4 Mb (Fig. 3) that covered at least 39 characterised genes (NCBI Bos taurus Annotation Release 105, UMD 3.1.1) including the two genes regarded as most potent candidates; *NCOA6* and *ACSS2*. The most significant marker for C8:0, C10:0, C12:0 and C14:0 was rs381037433 at 64,523,817 bp (p-values = 1.08e-18, 7.6e-16, 4.8e-14 and 5.2e-14, respectively), which is an intronic insertion (G/GA) in *phosphatidylinositol glycan anchor biosynthesis class U* (*PIGU*). The insertion had a frequency of 0.21 and was associated with a reduction of C8:0 level of 0.02 g/100 g milk fat. C6:0 was most significantly associated to rs41700742 at 64,648,620 bp (p-value = 6.8e-16), which is a synonymous SNP in *NCOA6*. LD (r^2^) between these two markers is 0.3.

Many other markers in the 63.5 to 65.4 Mb region displayed p-values and allele substitution effects similar to those of rs381037433. However, MAFs varied from 0.07 to 0.47. Haplotype analyses revealed that the least frequent allele of all these markers was present in one specific haplotype with a frequency of approximately 0.08 that spanned the entire 63.5 to 65.4 Mb region. For markers where the MAF was higher than 0.08, the least frequent allele was also found in other haplotypes. Hence, the LD (r^2^) among the most significant markers were generally low.

Only two non-synonymous SNPs were found among these top-ranking markers; rs383480158 in *peroxisomal membrane protein 4* (*PXMP4*) and rs446495267 in *PIGU*. Also, there were two 3′ UTR variants in *zinc finger protein 341* (*ZNF341*) and *ENSBTA00000000308*. However, neither of these genes have a function that can easily be related to milk fat synthesis. All other significant markers were either synonymous (i.e. not causing an amino acid shift) or positioned in non-coding regions such as introns and intergenic regions. This suggests a regulatory role also for this QTL. Results for all tested markers and trait combinations are shown in Supplementary Fig. S7 and Supplementary Table S3.

### Chromosome 17

**Figure 4:**
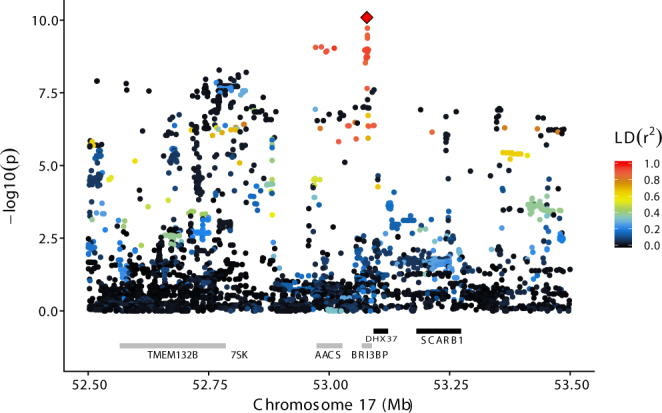
Results for BTA17 - C4:0 association analysis using imputed sequence variant data on BTA17, zoomed in on the region between 52.5 and 53.3 Mb. The ordinate provides −log_10_(p-value) for each marker – trait association, while the abscissa denotes marker position. The red diamond indicates the most significant marker for C4:0; rs477658921 at position 53,078,216 bp. Colouring indicates the level of LD (r^2^) between each marker and rs477658921. Gene annotation information (Ensembl Bos taurus annotation release 86) is shown with grey and black bars reflecting positive and negative strand orientations respectively.

In agreement with the GWAS analysis, imputed sequence variants on BTA17 were found to have a main effect on short C4:0 and C6:0 fatty acids. The most significant association was found for C4:0 and rs477658921 at 53,078,216 bp (Fig. 4). This is a 7-bp indel (GAAAGTGA/G) where the minor G allele (MAF = 0.08) was associated with an increase of C4:0 level of 0.05 g/100 g milk fat. This variant showed a lower significance against C6:0 (p-value = 1.3e-9) and no significance with other longer acids.

The rs477658921 indel is located within intron 1 of *BRI3 binding protein* (*BRI3BP*), which does not appear to be an especially good functional candidate gene, but it is also in close proximity to *AACS* at 52.97–53.03 Mb which may be involved in utilizing ketone body for fatty acid-synthesis. LD among the top-ranking markers was high (r^2^ higher than 0.84), indicating that the significance of the rs47765892 polymorphism on C4:0 could be a result of polymorphisms in or near *AACS*. As with BTA11, we found that the least frequent alleles of the most significant markers were contained within a haplotype with a frequency of 0.083 that spanned *AACS* and *BRI3BP*. All the top-ranking markers are either situated in introns of or outside these two genes and suggest a regulatory role of the QTL.

A second peak was detected at 51.49 Mb within the *zinc finger protein 280B* (*ZNF280B*). The LD between significant SNPs within this QTL region and the QTL embracing *AACS* and *BRI3BP* at 53.07 Mb is low which suggests that these are two different QTLs. The p-values of this second peak were approximately 1e-9 for all traits. Results for all tested markers and trait combinations are shown in Supplementary Fig. S8 and Supplementary Table S4.

### Chromosome 19

**Figure 5:**
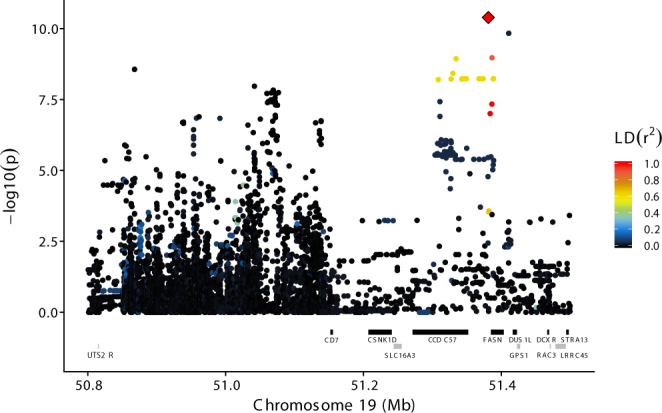
Results for BTA19 – C14:0 association analyses using imputed sequence variant data on BTA19, zoomed in on the region between 50.8 and 51.5 Mb. The ordinate provides −log_10_(p-value) for each marker – trait association, while the abscissa denotes marker position. The red diamond indicates the most significant marker for C14:0; rs109016955 at position 51,381,233 bp. Colouring indicates the level of LD (r^2^) between each marker and rs381037433. Gene annotation information (Ensembl Bos taurus annotation release 86) is shown with grey and black bars reflecting positive and negative strand orientations respectively.

The QTL on BTA19 was most strongly associated to C14:0 with significance levels decreasing for acids with shorter chain length and until it dropped below the significance threshold for C8:0 and shorter acids. The most significant marker for C14:0, C12:0 and C10:0 was rs109016955 at 51,381,233 bp (p-values = 4.1e-11, 7.5e-9 and 5.2e-9, respectively). This G/C SNP has a MAF of 0.04 where the minor C allele was associated with a reduction of C14:0 level of 0.14 g/100 g milk fat. It is situated ≈3,7 kb upstream of the transcription start site of *FASN* and annotated both as an upstream gene variant of *FASN* and as a 3′ untranslated region (3′ UTR) variant and a non-coding exon variant in various predicted transcript variants of *coiled-coil domain containing 57* (*CCDC57*). Similarly high significance levels were found for 24 variants situated either in introns of *CCDC57* and *FASN* or in the region between these two genes (Fig. 5). The MAF of all these markers were very low (0.03 to 0.05), and most of them showed only moderate LD with rs109016955 (Fig. 5). Results for all tested markers and trait combinations are shown in Supplementary Fig. S9 and Supplementary Table S5.

### Investigations of expression levels and transcripts

To verify that the candidate genes we detected from GWAS are present in the udder during lactation we isolated mRNA from somatic milk cells and measured their level of expression. Specifically, all genes in the region between 63.5 to 65.4 Mb on BTA13 were tested, along with candidates and, where appropriate, neighbour candidates on BTA11, 17 and 19. Expression levels in the form of mean normalised gene count can be found as Supplementary Table S10. *PAEP* was the most abundantly expressed gene of those found to be significant in the association analyses, with a mean count of approximately 444,000 reads. No reads were found for its neighbour *GLT6D1*. The QTL region on BTA13 contains at least 39 characterised genes (NCBI *Bos taurus* Annotation Release 105, UMD 3.1.1) of which 28 genes were found to be expressed in somatic milk cells. Mean expression levels varied from two to 5,407 normalised counts for these genes, with the highest expression found for *ACSS2*. *NCOA6* showed the eight highest expression level of these genes. On BTA17, both *AACS* and *BRI3BP* were expressed in the SMC, but expression level of *AACS* was much higher than for *BRI3BP*. *FASN* on BTA19 showed the second highest expression level of the studied genes with a normalised read count of ~29,000, which was approximately 233 times higher than the expression level of the neighbour *CCDC57*.

## Discussion

Understanding the role of genetic variation on fatty acid composition in bovine milk may reveal opportunities to produce superior raw product, and at the very least will improve our understanding of the genetics of fatty acid synthesis. Uniquely, our study combined data from 4.6 million FTIR recordings (FA composition phenotypes) representing 640,000 cows, with combined high-density genotyping and whole genome sequencing representing 1,811 bulls. This analysis allowed us to reveal a number of genetic variants associated with the synthesis of short- and medium-chained FAs C4:0 to C14:0.

We identified one gene, *PAEP*, which is a novel candidate gene in the context of fatty acid content, and several previously known candidate genes. Our results revealed that concentration of C4:0 was most strongly affected by *PAEP* on BTA11 and *AACS* on BTA17. The QTL on BTA13, which is most likely caused by *NCOA6* or alternatively *ACSS2*, seems to be related to the generation of longer chain length acids, while the *de novo*-synthesis of the longest chain length acid, C14:0, is most strongly affected by a polymorphism in or around *FASN* on BTA19.

A key condition for using phenotype data (FA composition) predicted from FTIR profiles in an association study is that individual acids can be predicted with a high degree of confidence. The effectiveness of mid-infrared spectroscopy to predict bovine milk fatty acid composition have been thoroughly discussed in a number of papers^12,15–17,20–24^. Inaccurate predictions and correlations among acids or between acids and other milk components may reduce the ability to identify true QTLs and determine exactly which fatty acids that are affected. We have previously reported that FAs with a concentration of 1% or higher are predicted with acceptable accuracies^12^. This finding was also reflected in the current study, where all the tested *de novo*-synthesized acids (i.e., C4:0 to C14:0) were present in concentrations above 1% and had prediction accuracies (cross-validated squared Pearson product-moment correlation coefficients; R^2^CV) ranging from 0.73 (C4:0) to 0.90 (C6:0 - C14:0) and hence were considered well predictable. An argument against using FTIR to predict FA composition is that the acids are correlated to total fat and the prediction merely reflects total fat rather than individual acids^20^. This correlation was accounted for in our two studies by presenting the fatty acid concentrations as percentages of total fat instead of as gram acid per unit of milk^21–23^. Soyeurt^21^ suggested that predictions were due to real absorbance of the acids if the calibration correlations were higher than the correlations between the acids and total fat. As reported in our previous study^12^, the squared correlation to total fat ranged between 0.001 and 0.012, indicating that the predicted concentrations are due to real absorbance values of these acids. A consequence of this normalization is that the prediction accuracies are expected to be lower than when FA concentrations are expressed as a quantity per unit of milk^21–23^, however with the exception of C4:0, the accuracies were found to be comparable to those obtained by milk-based models^12,21–23^. Although C4:0 was predicted with lower accuracy than the other FAs included in our study, our analysis detected two candidate genes with functions judged relevant for C4:0 content. Separating FAs with similar chain lengths such as C4:0 and C6:0 using an FTIR approach can be challenging since their chemical structure is relatively similar, however, the technology allowed us to identify two clearly different QTL profiles for C4:0 and C6:0 which, if the phenotype measurements were severely confounded together, would not be as distinct as they appear to be.

**Figure 6:**
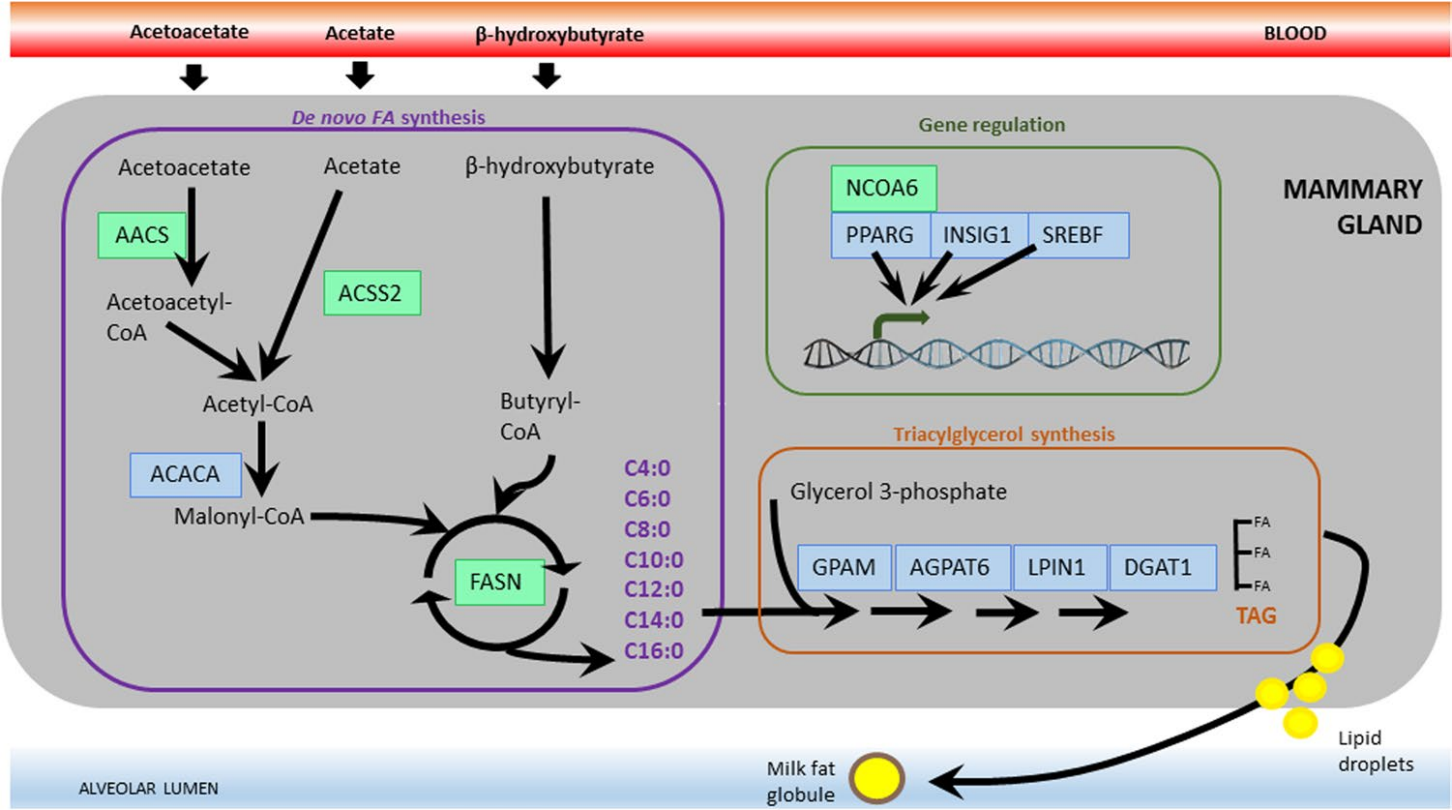
Illustration of the most relevant pathways and genes involved in *de novo* synthesis of short- and medium-chained fatty acids in the bovine mammary gland. Detected candidate genes are highlighted in green, whereas some additional well studied genes of high importance are shown in blue.

The genes highlighted as candidates for *de novo*-synthesis have, essentially, defined roles in bovine milk fat synthesis, and operate across the core pathways responsible for *de novo*-synthesis and triacylglycerol metabolism (Fig. 6). Early in *de novo*-synthesis, *ACSS2* facilitates the conversion of acetate to acetyl-CoA^25^. Alternatively, acetyl-CoA may be derived from acetoacetyl-CoA in the process beginning with the production of acetoacetate-CoA from acetoacetate by *AACS*^26^. Later, *FASN* oversees a process whereby palmitate (C16:0) is synthesised from acetyl-CoA and malonyl-CoA in a repeated, cyclic reaction. Importantly, intermediate length acids (C4:0 to C14:0) can leave the elongation cycle before the chain reaches full length. The entire lipid synthesis machine is regulated by a network of genes encoding transcription factors and nuclear receptors. One of these, *peroxisome proliferator-activated receptor gamma* (*PPARG*), is a well described transcriptional regulator affecting lipid storage^25,27^, while *NCOA6* (which is a ligand for *PPARG* and *PPARA*^28,29^) is a transcriptional coactivator enhancing the activity of, among other things, *PPARG*.

*PAEP* encodes the milk protein beta-lactoglobulin (β-LG) which is the major whey protein in bovine milk. Although the effect of *PAEP* on milk production traits including total fat yield and fat percentage has been well documented^30,31^, its influence on individual fatty acids is poorly understood. β-LG is found to bind both saturated and unsaturated FA *in vitro*^32^, which might suggest a function as an intracellular transporter of FAs. The B variant of the β-LG protein is commonly known to be less abundant than the A variant^31,33^, and it is unclear if the effect of *PAEP* on C4:0 found in our study is due to the polymorphisms causing the A and B β-LG variants or to regulatory sites affecting *PAEP* expression. Although the promoter region has been extensively studied, the causal polymorphism has not been identified due to an extensive level of LD between the two polymorphisms that produce the A and B protein variants and polymorphisms situtated within putative transcription factor binding sites^34–37^. The effect of this QTL on C4:0 could possibly be due to the combined influence of alterations in several sites simultaneously rather than to one specific SNP in one single site.

With regards to BTA13, previous studies have pointed towards *ACSS2*^38–40^ and *NCOA6*^12^ as positional and functional candidates. Due to high levels of LD among SNPs in the 2 Mb QTL region embracing these genes, our association analyses have expanded the BTA13 candidate list to include 39 characterised genes of which several have functions related to milk fat biosynthesis. This list also includes *E2F transcription factor 1* (*E2F1*) which is shown to regulate important genes involved in FA synthesis such as *ACSL1*, *FASN* and *PPARG*^41^, and *agouti signalling protein* (*ASIP*) which might regulate lipid metabolism in adipocytes^42^. However, the most significant markers detected by the association analyses were either found in non-coding regions or genes without known relevant functions in fat synthesis such as *PIGU*. Expression analyses revealed that 28 of the 39 genes were expressed in the bovine mammary gland during lactation. While *ACSS2* was distinctly more expressed than *NCOA6* in all samples (Supplementary Table S10), variants within and near *ACSS2* also showed a weaker association to the traits. Furthermore, since *NCOA6* contained variants that were among the top-ranking SNPs, we consider this gene to be the most promising positional candidate gene.

Our finding of an association between C4:0 and markers near *AACS* at ~53 Mb on BTA17 has not been reported in other GWA studies as far as we know. Li *et al*.^40^ and Duchemin *et al*.^43^ reported associations to markers on BTA17 in Chinese Holstein and Dutch Holstein-Friesian, respectively, but in other regions than *AACS*. This discrepancy may be explained by differences in study design (direct FA measurements compared to our study using DYDs estimated from millions of spectra from 640,000 cows) or the use of different breeds.

For BTA19, several authors have reported significant associations within or near *FASN* and the neighbouring gene *CCDC57*^40,44^. *CCDC57* is poorly characterised, and its putative role in milk fat synthesis is unknown. Medrano *et al*.^45^ reported that *CCDC57* was expressed in mammary tissues of a lactating cow with expression levels higher than that of *FASN*. This is in contrast with the results of the present paper, where *FASN* was expressed more than 200 times higher than *CCDC57. FASN* is an obvious candidate gene because of its known role in fat synthesis. *FASN* has been extensively studied in candidate gene studies for fat content in milk and adipose tissue^46–51^, but similarly to the present study, this has not yet resulted in a clear identification of a causal polymorphism.

Most genome scans performed in other cattle breeds than Norwegian Red cattle have reported strong associations between milk fatty acids and the genes *diacylglycerol acyltransferase 1* (*DGAT1*) on BTA14 and *stearoyl-coenzyme A desaturase 1* (*SCD*) on BTA26. *DGAT1* encodes an enzyme that catalyses the final stage of triacylglycerol synthesis^52^, while *SCD* on BTA26 is involved in the synthesis of monounsaturated FAs by introducing a double bond in the delta-9 position of C14:0, C16:0 and C18:0, primarily, thus producing the *cis*-9 variant of these acids^53^. No significant associations have been detected near these genes in any of our studies of Norwegian Red cattle. Resequencing of 147 widely used NR bulls revealed that all individuals were homozygous for the A variant of the DGAT1 K232A polymorphism (data not shown), suggesting that this variant is almost fixed in Norwegian Red cattle. The *SCD* polymorphism does segregate in our breed but was not significantly associated with any fatty acid neither in the present study nor in the previous study where a larger number of acids also including C14:1*cis*-9 and C16:1*cis*-9 were analysed^12^. However, these acids were poorly predicted by the FTIR approach^12^, which hampers the possibility to detect significant associations for these traits.

Imputation from HD-density to sequence level is heavily dependent upon MAFs and number of animals in the reference dataset^54^. In this study, 153 animals were whole genome sequenced and used as the imputation reference. When performed within breed, imputation for high-density genotypes to sequence has previously been shown to work acceptably with reference dataset of about 130^55^. We did not perform a cross validation procedure to test the expected accuracy in our dataset, but Beagle outputs a measure (AR^2^), defined as estimated squared correlation between most probable and true genotype, depending on the internally calculated uncertainty in the imputation model for each marker^56^. All markers with AR^2^ below 0.7 was filtered from our marker list before association analysis, as values above this threshold has shown to be a good indicator for reliable imputation accuracies^55,57,58^. Overall, mean AR^2^ was 0.92 for all sequence-level imputed variants, and 0.84 for variants with MAF below 0.05. AR^2^ was close to 1 for all variants found significant by the association analyses of sequence-level variants.

## Conclusions

Understanding of genes and polymorphisms controlling milk fat synthesis may reveal opportunities to tailor the fatty acid content and thereby improve the nutritional value and quality of dairy products. In this study we identified a set of positional candidate genes within milk fat synthesis pathways by combining dense genotyping and whole genome sequencing with high-throughput phenotypes for *de novo* synthesis of milk fatty acids. These genes were *PAEP* (on BTA11), *AACS* (BTA17), *NCOA6* or *ACSS2* (BTA13) and *FASN* (BTA19). Their roles in fatty acid synthesis were further supported by their expression levels in milk.

## Methods

### Ethics statement

All animals included in the study were Norwegian Red cattle, and experiments were conducted in accordance with the rules and guidelines outlined in the Norwegian Animal Welfare Act 2009, issued by the Norwegian Ministry of Agriculture and Food. Most data were generated as part of routine commercial activities outside the scope of that requiring formal committee assessment and ethical approval (as defined by the above guidelines).

### Estimation of bovine milk fat composition from FTIR spectroscopy data

Milk fat composition was estimated from FTIR spectroscopy data as described in Olsen *et al*.^12^, with some adjustments to number of spectra and animals used. In brief, 224 milk samples obtained from a feeding experiment and 659 samples from field sampling were analysed in parallel by FTIR and gas chromatography with flame ionization detector (GC-FID) reference analysis. FTIR spectra (regressors) were subsequently calibrated against GC-FID reference values (regressands) by using powered partial least squares regression (PPLSR^59^). Regressands were presented as percentages of GC-FID fatty acid values to total fat to reduce to a minimum value the correlation between the FA and total fat in milk samples. The calibration model was applied to a total of 4,619,737 infrared spectra from 640,304 cows sampled in the periods February to November 2007 and July 2008 to June 2014. The traits that were calibrated for in this study were C4:0, C6:0, C8:0, C10:0, C12:0 and C14:0.

A detailed description of the estimation of heritabilities and DYDs is given in in Olsen *et al*.^12^. In short, the estimation of heritabilities were performed on a reduced dataset of 2,209,486 profiles from 426,505 cattle with a pedigree of 716,753 animals using the DMU software version 6 release 5.1^60^. The data were analysed with the following mixed linear animal repeatability model:

1 $$Y=RY{M}_{i}+RP{L}_{j}+ht{d}_{k}+p{e}_{l}+{a}_{m}+{e}_{ijklm}$$

where RYM is the fixed effect of region (9 regions) by year and month of the test-day, with i ranging from 1 to 740; RPL is the fixed effect of region by lactation number by 10-day period in lactation of the test-day, with j ranging from 1 to 1,116; htd is the random effect of herd by test-day, with k ranging from 1 to 168,483; pe is the random permanent environmental effect of the cow on her repeated records, with l ranging from 1 to 426,505; a is a random additive genetic effect of the animal, with m ranging from 1 to 716,753; and e is a random residual effect.

DYDs for the GWAS were then estimated using the 4,619,737 spectra for the full dataset of 640,304 cows with a pedigree of 999,470 animals as the sire averages of daughters’ predicted FA compositions, which were each corrected for her fixed effects, non-genetic random effects and half of her dam’s genetic effect^12^.

The concentration of each fatty acid together with the accuracy of prediction (in the form of cross-validated squared Pearson product-moment correlation coefficients; R^2^CV) and heritabilities were as reported in Olsen *et al*.^12^. Mean concentration ranged from 1.48% of total fat for C8:0 to 11.21% of total fat for C14:0. R^2^ ranged from 0.73 for C4:0 to 0.91 for C8:0, C10:0 and C12:0, while heritabilities ranged from 0.11 for C14:0 to 0.35 for C4:0.

### Construction of a dense SNP dataset

Genotypes for the studied animals were available from other projects and the routine genotyping performed by Geno Breeding and AI Association. DNA was extracted from semen samples of bulls and from blood samples of cows using standard phenol-chloroform-based protocols. The bulls were genotyped on at least one of four different platforms in order to make a genome-wide high-density SNP dataset for the association analyses; the Affymetrix 25 K SNP array (Affymetrix, Santa Clara), a custom Affymetrix 50 K SNP array, the Illumina 54 K BovineSNP50 BeadChip (Illumina, San Diego) and the 777 K Illumina BovineHD Genotyping BeadChip (Illumina, San Diego).

Imputation was done in a step-wise manner, were the 25 K Affymetrix dataset was imputed to the custom 50 K Affymetrix density, and then the combined Illumina 54 K and Affymetrix 50 K dataset were imputed to 777 K. The Affymetrix 50 K reference counted 5,009 NR animals and the Illumina 777 K reference consisted of 750 widely used AI bulls. Imputation was done using Beagle version 4.1^18^, with effective population size (Ne) set to 200 and number of phasing iterations (niterations) set to 20. Remaining parameters were set to default. Map positions were based on the UMD 3.1 reference assembly^61^.

For each imputation step, the following quality control of the markers was applied: Variants with MAF less than 0.01 and Hardy-Weinberg Equilibrium p-values less than 1e-7 were filtered. Animals with more than 10% Mendelian errors were removed from the dataset, and all remaining genotypes with Mendelian errors were set to missing and later imputed. Markers and animals with a call rate below 95% were removed. Markers on sex chromosomes were discarded. For each step, the imputation quality was tested using 5-fold cross validation. Markers with discordance between true and imputed genotypes above 10% were removed, as these markers are likely to be misplaced in the reference assembly^62^. SNPs on unplaced scaffolds and sex chromosomes were also discarded from the dataset.

A total of 2,434 genotyped AI bulls were considered for the initial 777 k GWAS analysis. After filtering bulls with less than 20 daughters, the dataset contained 1,811 bulls with imputed genotypes for the 777 K Illumina BovineHD BeadChip. Of the 1,811 bulls, 57 bulls had genotypes imputed from the Affymetrix 25 K array, 237 were imputed from the custom Affymetrix 50 K SNP array, 1,113 animals from the Illumina 54 K BeadChip and 404 were already genotyped on the 777 k Illumina BovineHD BeadChip. The resulting dataset consisted of 1,811 bulls with trait data in the form of DYDs based on 20 or more daughters for the relevant FAs and with genotypes for 609,361 SNPs distributed on all 29 autosomes.

### Whole-genome sequencing and variant calling

Whole-genome sequencing data were obtained from 153 animals (132 AI bulls and 31 cows) as described in Olsen *et al*.^63^. The AI bulls were selected based on maximum number of daughters in production and by ensuring an even contribution to the population structure of Norwegian Red cattle, by manually examining the recorded pedigree. Animals were sequenced to an average coverage of 9 × using Illumina sequencing (Illumina, San Diego). All reads were aligned against UMD 3.1 using BWA-mem version 0.7.10^64^. Variant calling was done with FreeBayes version 1.0.2^65^. Missing genotypes in the resulting Variant Call Format (VCF) file were imputed and phased using Beagle version 4.1^18^. This phased dataset was used as a reference panel for imputing the 1,811 animal high-density panel to full sequence with the Beagle software using the same imputation parameters as described before except that expected allele miscall rate (err) were set to 0.01. In a final filtering step, variants with minor allele frequency above 0.02 were kept. Also, variants with Beagle’s reported allelic R^2^ (AR^2^) below 0.7 were filtered, as this has been shown to be a robust and reliable threshold for filtering of imputed sequence variants^56–58^. The raw, unfiltered VCF-file were kept for future reference.

### Genotyping of cows

The 36 cows used for the RNA sequencing were also genotyped on the Illumina BovineSNP50 BeadChip (54 K, Illumina, San Diego). Blood samples were collected by certified personnel, and DNA extraction and genotyping on the Illumina BovineSNP50 BeadChip (54 K, Illumina, San Diego) were performed according to the manufacturer’s protocol. Genotypes were quality checked and imputed to sequence density as described above.

### Genome-wide association studies

A single-marker genome-wide association analysis was performed for the fatty acids C4:0 to C14:0, and 609,361 genome-wide distributed SNPs. This analysis was conducted with the GCTA software^66^ for computational feasibility. A mixed linear model association analysis was performed with the –mlma-loco option of GCTA. The model fitted to the performance information for each trait and each SNP was:

2 $$DYD=a+bx+{g}^{-}+e$$

were DYD is the performance of the bull, a is the mean term, b is the fixed additive effect of the candidate SNP to be tested for association, x is the SNP genotype indicator variable coded as 0, 1 or 2, g^−^ is the random polygenic effect, i.e. the accumulated effect of all SNPs except those on the chromosome where the candidate SNP is located, and e is the residual. The var(g^−^) will be re-estimated each time when a chromosome is excluded from calculating the genomic relationship matrix. The chromosome-wide significance level was set at p = 1e-5 which is a default value from qqman^67^. The genome-wide significance level was set at (0.05/609,361*6) = 1.37e-8, corresponding to a nominal type I error rate of 0.05 and Bonferroni correction for 609,361 markers and 6 traits.

### Re-analyses of selected regions on sequence-level variants

All sequence-level polymorphisms that passed quality control and were situated in the QTL regions detected by the GWAS were analysed using the ASReml package version 2.0^68^. ASReml were selected for this step since it allowed us to weight the DYDs by number of daughters as well as to use genotype dosage data as input.

Analysed regions and traits were 100 to 107 Mb on BTA11 (C4:0), 60 to 70 Mb on BTA13 (C6:0 to C14:0), 20 to 60 Mb on BTA17 (C4:0 and C6:0) and 45 to 55 Mb on BTA19 (C10:0 to C14:0).

The model that was fitted to the information on performance for each trait – marker combination was:

3 $${\bf{DYD}}={\bf{1}}\mu +{\bf{X}}{\rm{b}}+{\bf{Za}}+{\bf{e}}$$

where **DYD** is the vector of bull performances weighed by the number of daughters, **1** is a vector of ones, *μ* is the overall mean, **X** is a vector of marker genotypes coded as a decimal number between 0 and 2 depending on the estimated dosage of the alternate allele (as reported by Beagle 4.1), b is the fixed effect of the marker, **Z** is an incidence matrix relating phenotypes to the corresponding random polygenic effects, **a** is a vector of random polygenic effects, and **e** is a vector of residual effects. Genetic and residual variances were estimated from the data. **a** was assumed to follow a normal distribution $\, \sim N({\bf{0}},{\bf{A}}{\sigma }_{{\bf{A}}}^{2})$ where **A** is the relationship matrix derived from the pedigree, and ${{\rm{\sigma }}}_{{\bf{A}}}^{2}$ is the additive genetic variance. **e** was assumed to follow a normal distribution $\, \sim N({\bf{0}},{\bf{I}}{\sigma }_{{\bf{e}}}^{2})$ where ${{\rm{\sigma }}}_{e}^{2}$ is the residual variance. Association analysis was performed for each individual marker. Since ASReml does not output p-values for the marker effect automatically, these were calculated from the F statistics for the conditional sum of squares, the numerator degrees of freedom and the denominator degrees of freedom with the R function pf() from the stats package version 3.4.0.

### Haplotype analyses

Pairwise LD measurements (r^2^) were estimated and haplotypes were identified for the top ranking markers within the relevant QTL regions using the Haploview 4.2 software^69^ on phased genotypes. Haplotypes were defined by Haploview according to the confidence intervals strategy^70^ or the four gamete rule^71^.

### RNA isolation, sequencing and read mapping

Gene expression levels were obtained using read counts from mRNA isolated from somatic milk cells (SMC) of 36 cows from the research facilities at the Norwegian University of Life Sciences, Aas, Norway. Pedigree information was used to avoid selection of close relatives. The cows were part of a research herd at our University. All milk samples were collected approximately 50 days (range 47 to 55) after calving. This sampling period was chosen since it coincides approximately with peak expression of several relevant genes involved in bovine milk fat synthesis including *FASN*^25^ and also with the peak of synthesis and import of FAs in bovine milk^25^ and the top of the lactation curve of Norwegian Red cows^72^. The cows were in different parities due to the limited size of the research herd. All animals were fed an equal standard diet.

Milk is excreted by the mammary epithelial cells (MEC) lining the inside of the udder, which are subject to turnover and shed into the milk and therefore represent a proportion of the somatic cells found in milk^73^. Cánovas *et al*.^74^ found that compared to other sources (e.g. mammary gland tissue, laser dissected MEC), the quality of the total RNA extracted from the SMC was high. Moreover, the expression of genes investigated in SMC derived material was highly correlated with the expression observed in laser-dissected MEC. Several studies have confirmed the usefulness of this method^73,75,76^.

Milk samples were collected manually 2–3 hours after milking to maximise the amount of viable cells present in the milk. Teats were cleaned with water followed by 70% ethanol before milking by hand, and 2 × 50 ml milk from each animal was collected in Falcon tubes. Samples were stored on ice immediately after collection and centrifuged at 4 °C for 10 min at 2,300 g within 1.5 hours to collect cells in the bottom of tubes. After centrifugation, most of the fat layer was removed with a clean pipette tip and supernatant decanted. Each pellet was dissolved in 4 ml 1xPBS by pipetting up and down. The liquid was transferred to a new 50 ml Falcon tube. Samples were centrifuged at 4 °C for 10 min at 2,300 g and supernatant decanted. Cell pellets were dissolved in 1 ml Trizol (Qiagen), and cells were lysed by pipetting up and down. Samples were stored in −80 °C until RNA extraction with Qiagen RNeasy Plus Universal Tissue Mini Kit (Qiagen) according to the manufacturer’s protocol. RNA concentrations and quality were measured with a NanoDrop8000 spectrophotometer (Thermo Fisher Scientific) and Agilent RNA 6000 assay on Agilent BioAnalyzer 2100 (Agilent Technologies), respectively. All samples had an RNA integrity number (RIN) between 6.6 and 9.2. Samples were prepared for paired-end sequencing (2 × 150 bp) using the Illumina® TruSeq® stranded mRNA library preparation kits and sequenced by the Norwegian Sequencing Centre (www.sequencing.uio.no) using an Illumina HiSeq. 3000 platform.

Before mapping, raw read quality were assessed using fastQC version 0.11.5 https://www.bioinformatics.babraham.ac.uk/projects/fastqc/), Illumina adaptors were removed, and the sequences were quality-trimmed using cutadapt^77^. Cutadapt was set to cut adaptors with a minimum overlap length of 8 and low-quality 3′ ends were removed by setting a quality threshold of 20 (phred quality + 33). An index of the UMD 3.1 reference genome was built, and reads were aligned to the reference using STAR version 2.3.1^78^. Sorting, indexing and conversion to the BAM file format (the compressed binary version of a SAM file) of the resulting SAM files were completed using SAMtools version 1.3^79^. The code for the described RNAseq mapping method is available as part of a bash-script pipeline (version 1.1.0) found at https://gitlab.com/fabian.grammes/RNAseq-analysis/.

### Variant annotations

All variants were annotated using the web version of Ensembl Variant Effect Predictor^80^ based on the Ensembl Bos taurus annotation release 86.

### Availability of data

DNA and RNA sequence data will be submitted to the European Nucleotide Archive, http://www.ebi.ac.uk/ena. Phenotype and genotype data are available only upon agreement with Geno Breeding and AI Organization (http://www.geno.no).

## Electronic supplementary material

Supplementary Information

Supplementary Table S1

Supplementary Table S2

Supplementary Table S3

Supplementary Table S4

Supplementary Table S5

Supplementary Table S10
