# Supplementary material for: Unravelling genetic variation underlying de novo-synthesis of bovine milk fatty acids
Source: Sci Rep. 2018 Feb 1;8:2179. doi: 10.1038/s41598-018-20476-0 (PMC5794751; doi:10.1038/s41598-018-20476-0)
Supplement: Supplementary file 1 — Supplementary Information [file 41598_2018_20476_MOESM1_ESM.pdf]

SUPPLEMENTARY INFORMATION FROM:

Unravelling genetic variation underlying *de novo*-synthesis of bovine milk fatty acids.

Tim Martin Knutsen[1]; Hanne Gro Olsen[1,*]; Valeria Tafintseva[2], Morten Svendsen[3]; Achim Kohler2; Matthew Peter Kent[1]; Sigbjørn Lien[1].

[1] Centre for Integrative Genetics (CIGENE), Department of Animal and Aquacultural Sciences (IHA), Faculty of Life Sciences (BIOVIT), Norwegian University of Life Sciences (NMBU), PO Box 5003, Ås, NORWAY

[2] Faculty of Science and Technology (RealTek), Norwegian University of Life Sciences (NMBU), PO Box 5003, Ås, NORWAY

[3] Geno Breeding and AI Association, N-1432 Ås, Norway

Corresponding author information:

Hanne Gro Olsen

Centre for Integrative Genetics (CIGENE)

Department of Animal and Aquacultural Sciences (IHA)

Faculty of Life Sciences (BIOVIT)

Norwegian University of Life Sciences (NMBU)

PO Box 5003

N-1432 Ås

Norway

Phone: + 47 67232653

e-mail: hanne-gro.olsen@nmbu.no

Supplementary Table S1. Table showing GWAS results. All significant ($p<1e-5$) marker – trait combinations from the GWA analysis.

Supplementary Table S2. Results for single-marker association analyses ($p<1e-5$) of C4:0 on imputed sequence data in the region between 100 and 107 Mb on BTA11.

Supplementary Table S3. Results for single-marker association analyses ($p<1e-5$) of C6:0 to C14:0 on imputed sequence data in the region between 60 and 70 Mb on BTA13.

Supplementary Table S4. Results table for single-marker association analyses ($p<1e-5$) of C4:0 and C6:0 on imputed sequence data in the region between 20 and 60 Mb on BTA17.

Supplementary Table S5. Results for single-marker association analyses ($p<1e-5$) of C6:0 to C14:0 on imputed sequence data in the region between 45 and 55 Mb on BTA19.

Supplementary Figure S6. Results for single-marker association analyses of C4:0 using imputed sequence variant data in the region between 100 and 107 Mb on BTA11. The ordinate provides $-\log_{10}$(p-value) for each marker – trait association, while the abscissa denotes marker position.

Supplementary Figure S7. Results for single-marker association analyses of C6:0 to C14:0 using imputed sequence variant data in the region between 60 and 70 Mb on BTA13. The ordinate provides $-\log_{10}$(p-value) for each marker – trait association, while the abscissa denotes marker position.

Supplementary Figure S8. Results for single-marker association analyses of C4:0 to C14:0 using imputed sequence variant data in the region between 45 and 55 Mb on BTA17. The ordinate provides $-\log_{10}$(p-value) for each marker – trait association, while the abscissa denotes marker position.

Supplementary Figure S9. Results for single-marker association analyses of C4:0 to C14:0 using imputed sequence variant data in the region between 45 and 55 Mb on BTA19. The ordinate provides $-\log_{10}$(p-value) for each marker – trait association, while the abscissa denotes marker position.

Supplementary Table S10.

Mean normalized read count with standard deviation (SD) for the genes in the QTL regions on BTA11, 13, 17 and 19.

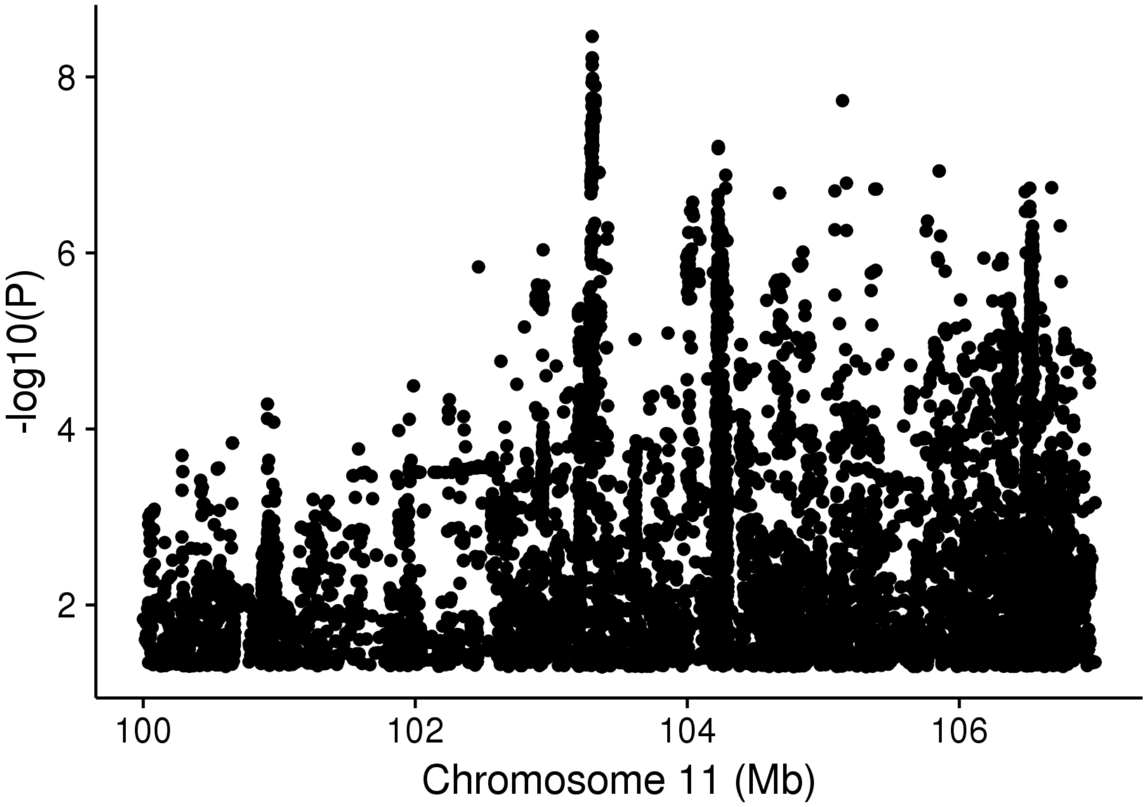

Figure S6.

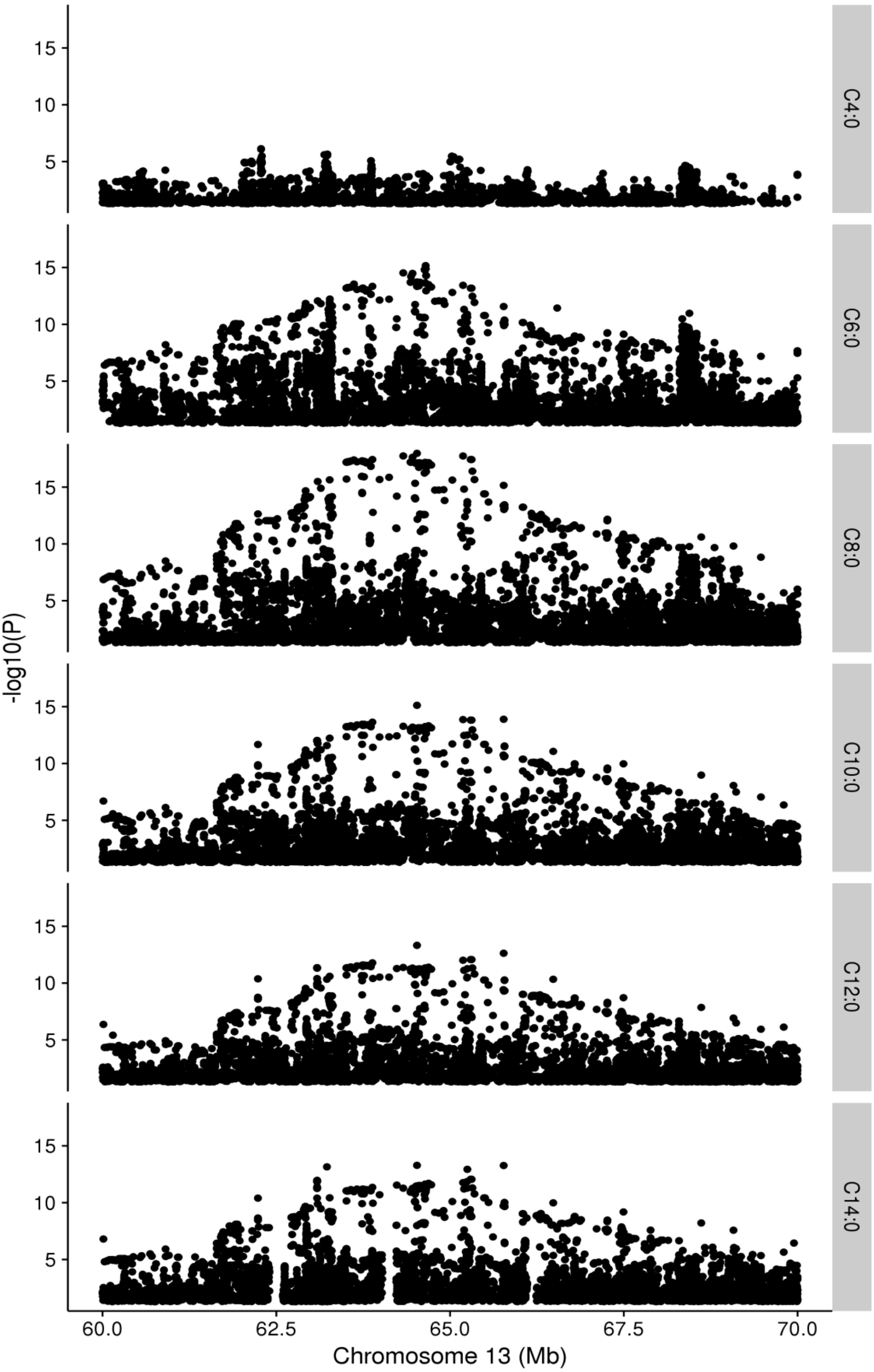
C4:0
C6:0
C8:0
C10:0
C12:0
C14:0
-log10(P)
15
10
5
60.0
62.5
65.0
67.5
70.0
Chromosome 13 (Mb)

Figure S7

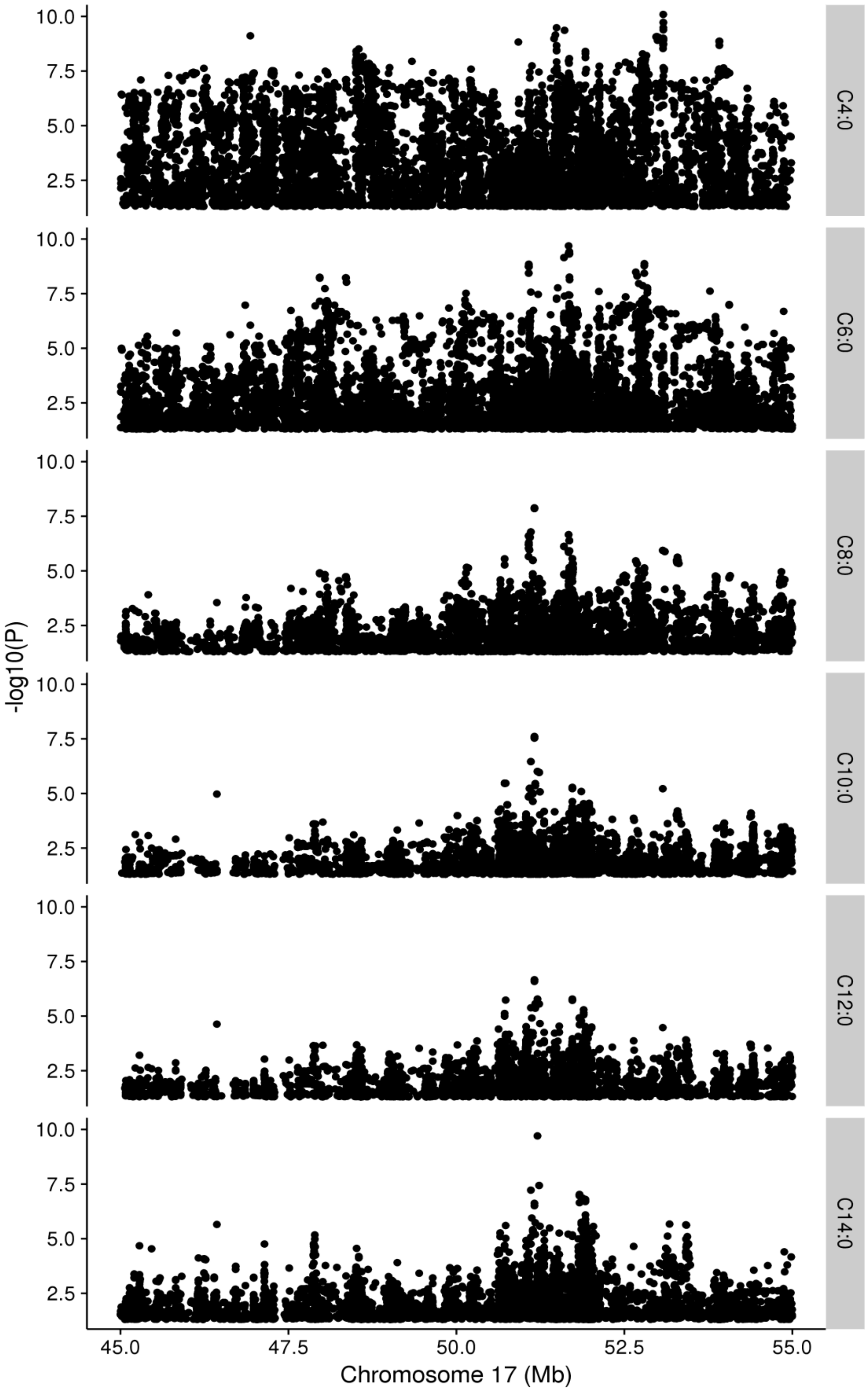

10.0
7.5
5.0
2.5
-log10(P)
C4:0
C6:0
C8:0
C10:0
C12:0
C14:0
45.0
47.5
50.0
52.5
55.0
Chromosome 17 (Mb)

Figure S8

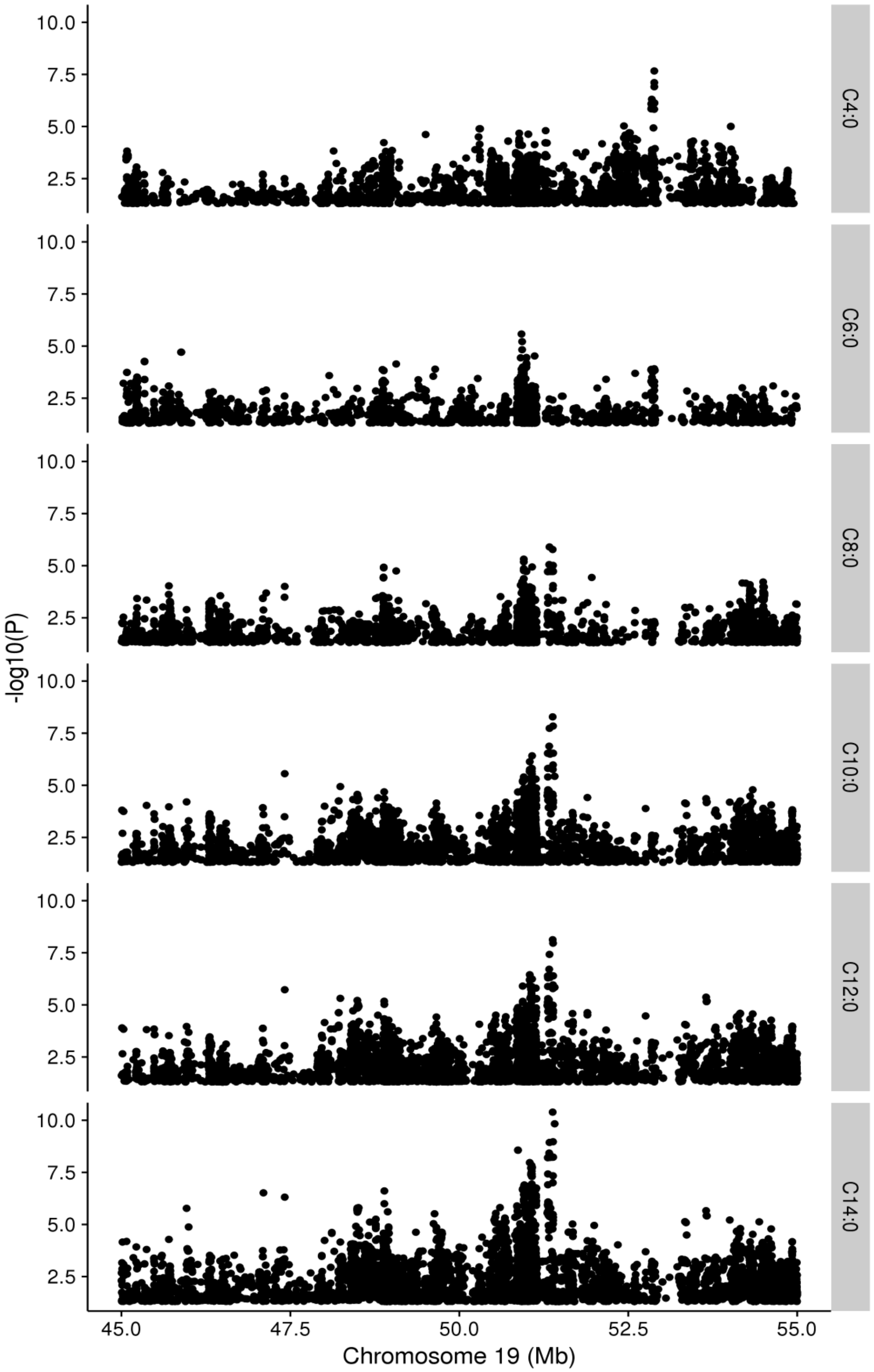
C4:0
C6:0
C8:0
C10:0
C12:0
C14:0
10.0
7.5
5.0
2.5
-log10(P)
45.0
47.5
50.0
52.5
55.0
Chromosome 19 (Mb)

Figure S9
